# Data on health risk assessment to the nitrate in drinking water of rural areas in the Khash city, Iran

**DOI:** 10.1016/j.dib.2018.11.007

**Published:** 2018-11-03

**Authors:** Majid Radfard, Massuomeh Rahmatinia, Hamidreza Tabatabaee, Hamed Solimani, Amir Hossein Mahvi, Abolfazl Azhdarpoor

**Affiliations:** a Department of Environmental Health Engineering, School of Public Health, Shiraz University of Medical Sciences, Shiraz, Iran; b Student Research Committee, School of Public Health, Shahid Beheshti University of Medical Sciences, Tehran, Iran; c Department of Epidemiology and Statistics, Shiraz University of Medical Sciences, Shiraz, Iran; d Department of Environmental Health Engineering, School of Public Health, Tehran, Tehran, Iran; e Health Research Center, Lifestyle, Institute, Baqiyatallah University of Medical Sciences, Tehran

**Keywords:** Risk assessment, Nitrate, Drinking water, Khash, Iran

## Abstract

The main objective of this data was determination of the nitrate concentration and its health risk assessment in the drinking water resources. In the aim of this data article a number of 30 samples of nitrate concentration in drinking water resources were collected from villages of the Khash city, Sistan and Baluchistan province, Iran. The nitrate concentration was measured using a Spectrophotometer accordance standard methods for examination of water and wastewater. Data indicated that nitrate concentration in drinking water ranged from 6 to 35 mg/L (average 16.083 mg/L). The mean EDI values for nitrate in different groups of infants, children, teenagers and adults were 0.1287, 0.9114, 0.6433 and 0.5155 mg/Kg, respectively. The findings of data showed that HQ value was less than 1 in 96% of samples in age groups of infants, children, teenagers and adults.

**Specifications table**

**Table:** 

Subject area	Water quality and risk assessment
More specific subject area	Nitrate in drinking water
Type of data	Table and Figure
How data was acquired	All water samples were analyzed using a UV-visible Spectrophotometer (DR/5000, USA) according to standard methods for examination water and wastewater.
Data format	Raw, Analyzed
Experimental factors	Water samples were taken from rural water resources.
Experimental features	Determine the concentration levels of nitrate
Data source location	Khash region of Sistan and Baluchistan province, Iran
Data accessibility	The data are available with this article

**Value of data**

• The data showed that the nitrate concentration in all of water samples was lower than the maximum permissible limits (50 mg/L) according to WHO guideline and Iran standards.
• Rural water sources, due to the lack of sewage collection systems can be one of the sources of nitrate absorption in the body and cause methemoglobinemia (blue baby), especially in children. Also, nitrate is used mainly in inorganic fertilizers. It is also used as an oxidizing agent and in the production of explosives, and purified potassium nitrate is used for glass making, so nitrate can reach both surface water and groundwater (by infiltration of the nitrogen) as a consequence of agricultural activity. Hence its risk assessment can be useful in preventing methemoglobinemia.
• The data indicated that HQ value was more than one for age group of children only in one sampling areas, so should be selected a suitable resource of drinking water for this age group.

## 1 Data

**Fig. 1:**
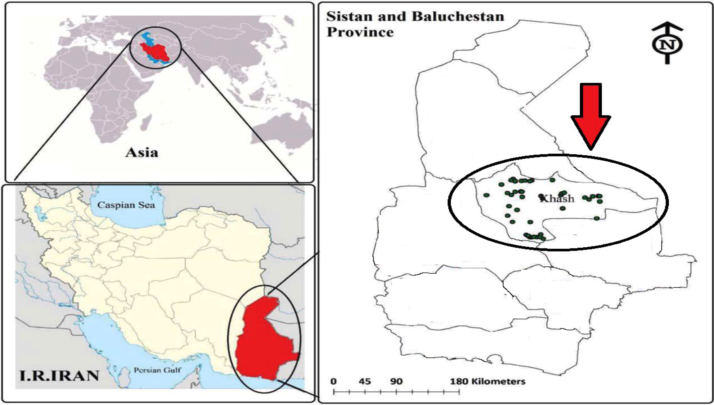
Location of nitrate sampling points in drinking water resources of the Khash city.

**Fig. 2:**
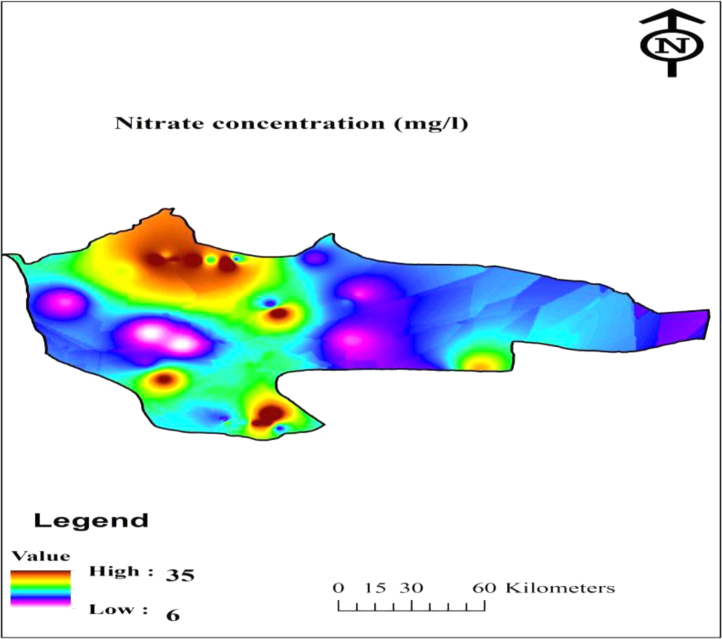
Geological distribution of nitrate in studied area.

Table 1 shows the constants used in calculating the nitrate risk assessment in water samples. Nitrate concentration and nitrate estimated daily intake (EDI) and hazard quotient (HQ) of nitrate for the drinking water samples have been indicated in Table 2. Also, location of water sampling in the research area and Geological distribution of nitrate in Khash area has been indicated in Fig. 1, Fig. 2.

**Table 1:** Constants used to in the present data for the nitrate risk assessment in water drinking [1], [2], [3], [4], [5], [6], [7], [8].

Parameter	Risk exposure factors	Values for groups				Unit
		Infants	Children	Teenagers	Adults	
Nitrate	C_f_	–	–	–	–	mg/L
	C_d_	0.08	0.85	2	2.5	Liter/day
	B_w_	10	15	50	78	kg
	RfD	1.6	1.6	1.6	1.6	mg/kg.day

**Table 2:** Nitrate concentration, estimated daily intake and hazard quotient for the four populations of water consumers.

No.s	Nitrate		EDI		Adults	Infants	HQ		
	concentration	Infants	Children	Teenagers			Children	Teenagers	Adults
1	11.500	0.0920	0.6517	0.4600	0.3686	0.0575	0.4073	0.2875	0.2304
2	26.000	0.2080	1.4733	1.0400	0.8333	0.1300	0.9208	0.6500	0.5208
3	19.000	0.1520	1.0767	0.7600	0.6090	0.0950	0.6729	0.4750	0.3806
4	18.500	0.1480	1.0483	0.7400	0.5929	0.0925	0.6552	0.4625	0.3706
5	6.500	0.0520	0.3683	0.2600	0.2083	0.0325	0.2302	0.1625	0.1302
6	8.500	0.0680	0.4817	0.3400	0.2724	0.0425	0.3010	0.2125	0.1703
7	6.000	0.0480	0.3400	0.2400	0.1923	0.0300	0.2125	0.1500	0.1202
8	24.500	0.1960	1.3883	0.9800	0.7853	0.1225	0.8677	0.6125	0.4908
9	16.000	0.1280	0.9067	0.6400	0.5128	0.0800	0.5667	0.4000	0.3205
10	27.000	0.2160	1.5300	1.0800	0.8654	0.1350	0.9563	0.6750	0.5409
11	25.000	0.2000	1.4167	1.0000	0.8013	0.1250	0.8854	0.6250	0.5008
12	35.000	0.2800	1.9833	1.4000	1.1218	0.1750	1.2396	0.8750	0.7011
13	16.000	0.1280	0.9067	0.6400	0.5128	0.0800	0.5667	0.4000	0.3205
14	11.500	0.0920	0.6517	0.4600	0.3686	0.0575	0.4073	0.2875	0.2304
15	26.500	0.2120	1.5017	1.0600	0.8494	0.1325	0.9385	0.6625	0.5308
16	8.500	0.0680	0.4817	0.3400	0.2724	0.0425	0.3010	0.2125	0.1703
17	11.500	0.0920	0.6517	0.4600	0.3686	0.0575	0.4073	0.2875	0.2304
18	11.500	0.0920	0.6517	0.4600	0.3686	0.0575	0.4073	0.2875	0.2304
19	9.000	0.0720	0.5100	0.3600	0.2885	0.0450	0.3188	0.2250	0.1803
20	8.000	0.0640	0.4533	0.3200	0.2564	0.0400	0.2833	0.2000	0.1603
21	18.500	0.1480	1.0483	0.7400	0.5929	0.0925	0.6552	0.4625	0.3706
22	20.500	0.1640	1.1617	0.8200	0.6571	0.1025	0.7260	0.5125	0.4107
23	7.000	0.0560	0.3967	0.2800	0.2244	0.0350	0.2479	0.1750	0.1402
24	10.500	0.0840	0.5950	0.4200	0.3365	0.0525	0.3719	0.2625	0.2103
25	11.000	0.0880	0.6233	0.4400	0.3526	0.0550	0.3896	0.2750	0.2204
26	25.500	0.2040	1.4450	1.0200	0.8173	0.1275	0.9031	0.6375	0.5108
27	15.000	0.1200	0.8500	0.6000	0.4808	0.0750	0.5313	0.3750	0.3005
28	14.000	0.1120	0.7933	0.5600	0.4487	0.0700	0.4958	0.3500	0.2804
29	25.500	0.2040	1.4450	1.0200	0.8173	0.1275	0.9031	0.6375	0.5108
30	9.000	0.0720	0.5100	0.3600	0.2885	0.0450	0.3188	0.2250	0.1803
Mean	16.083	0.1287	0.9114	0.6433	0.5155	0.0804	0.5696	0.4021	0.3222
Max	35.000	0.2800	1.9833	1.4000	1.1218	0.1750	1.2396	0.8750	0.7011
Min	6.000	0.0480	0.3400	0.2400	0.1923	0.0300	0.2125	0.1500	0.1202
SD	7.778	0.0622	0.4408	0.3111	0.2493	0.0389	0.2755	0.1945	0.1558

## 2 Experimental design, materials and methods

### 2.1 Description of study area

The Khash city is located in Sistan and Baluchistan Province, Iran in the latitudes of 28°13׳N and longitudes of 61°13׳E. According to the demographic information of Iran, this city is populated with almost 173,821 with an area 19.376 km^2^. This area has a warm and dry climate and the highest and lowest air temperatures are 37 °C and −7 °C, respectively [9], [10], [11].

### 2.2 Determination of nitrate concentration in drinking water resources

The samples were collected in the middle of the day and then transferred to chemical laboratory in a cool box immediately. Sampling was done with one‑liter polyethylene bottles which were immersed in nitric acid for 24 h, then washed with 10 percent HCL and finally washed with distilled water. It is necessary to be mentioned that before the collection of the samples, sampling containers had been rinsed at least three times with water. In the laboratory the nitrate ions in the samples were measured using the SPADN method at a wavelength of 500 nm by HACH (spectrophotometer DR 5000 Company, USA) [12], [13], [14], [15], [16], [17], [18], [19], [20], [21], [22], [23], [24].

### 2.3 Risk assessment of nitrate

The most important concern is the health effects of exposure to high concentrations of nitrate due to the occurrence of methemoglobinemia and nitrosamines. Therefore, health effects should be evaluated to prevent undesirable health effects [1], [2], [3], [4]. So, the quantitative health risk assessment of nitrate through consumption of drinking water was estimated in rural population of Khash city, Sistan and Baluchistan province. For this aim, water samples were collected from villages of Khash city. Then, population were divided into four age groups based on physiological and behavioral differences as fallow: infants (<2 years), children (2 to <6 years), teenagers (6 to <16 years) and adults (≥ 16 years). The daily exposure to nitrate was calculated in these groups using Eq. (1) [2], [8]:

(1) $$EDI=\frac{C_{f}\times C_{d}}{B_{w}}$$

EDI: Estimation of daily nitrate consumption (mg/kg)
C_f_: Nitrate concentration in drinking water (mg/L)
C_d_: Average daily drinking water intake
B_w_: Body weight (kg)

Then hazard quotient (HQ) was evaluated to predict the non-carcinogenic risk of exposure to nitrate using Eq. (2).

(2) $$HQ=\frac{EDI}{RFD}$$

EDI: Estimated daily intake (mg/kg d)
RFD: Reference dose

The reference dose of nitrate (1.6 mg kg^−1^ d^−1^) was according to the integrated Risk Information System, USEPA. A value HQ more than one indicates a significant risk level of non-carcinogenic effects.
