# Supplementary material for: Data on health risk assessment to the nitrate in drinking water of rural areas in the Khash city, Iran
Source: Data Brief. 2018 Nov 3;21:1918–23. doi: 10.1016/j.dib.2018.11.007 (PMC6260370; doi:10.1016/j.dib.2018.11.007)
Supplement: Supplementary file 1 — Supplementary material [file mmc1.docx]

noConflict of Interest Form
